# Actores del cuidado en itinerarios terapéuticos de personas mayores sobrevivientes al COVID-19 en Chile

**DOI:** 10.1590/0102-311XES161324

**Published:** 2025-04-28

**Authors:** Catalina Arteaga-Aguirre, Paulina Osorio-Parraguez, Constanza Biscarra-Mc-Naughton, Alejandra Fuentes-García, Ignacia Navarrete, María Gracia Salamanca-Garcia

**Affiliations:** 1 Departamento Sociología, Universidad de Chile, Santiago, Chile.; 2 Departamento de Antropología, Universidad de Chile, Santiago, Chile.; 3 Universidad de Chile, Santiago, Chile.; 4 Facultad de Medicina, Universidad de Chile, Santiago, Chile.

**Keywords:** Ruta Terapéutica, Envejecimiento, Cuidadores, COVID-19, Therapeutic Itinerary, Aging, Caregivers, COVID-19, Itinerário Terapêutico, Envelhecimento, Cuidadores, COVID-19

## Abstract

En este artículo se describen y analizan diferentes actores del cuidado presentes en itinerarios terapéuticos de personas mayores contagiadas y sobrevivientes al COVID-19, en diversos territorios de Chile. La estrategia metodológica fue cualitativa con enfoque etnográfico, centrada en itinerarios terapéuticos de personas mayores de 75 y más sobrevivientes de COVID-19, durante los años 2020 y 2021. El trabajo de campo se desarrolló en dos regiones, analizando experiencias diferenciales en procesos de atención y cuidados en territorios urbanos y rurales. Se aplicó análisis de contenido narrativo y construcción de relatos etnográficos, sobre los itinerarios terapéuticos de personas mayores y sus cuidadores/as. Los resultados muestran la diversidad y complejidad de actores involucrados, en la trayectoria de cuidados en contexto de contagio por COVID-19: familia, vecinos/as, amistades, sistemas público y privado de salud, entre otros, así como distintas modalidades en que estos se articulan para el cuidado de las personas mayores. Se destaca la articulación de actores públicos, privados y comunitarios; formales e informales, durante el proceso.

## Abstract (en)

This article describes and analyzes different care actors in therapeutic itineraries of older adults diagnosed with COVID-19 and survived it in different territories of Chile. A qualitative methodological strategy with an ethnographic approach was employed, focusing on the therapeutic itineraries of adults aged 75 years or older, survivors of COVID-19, from 2020 to 2021. Fieldwork was carried out in two regions, analyzing differential experiences in care processes in urban and rural areas. Narrative content analysis and ethnographic story construction about the therapeutic itinerary of older adults and their caregivers were applied. The results show the diversity and complexity of the actors involved in the trajectory of care in the context of COVID-19 infection: family, neighbors, friends, public and private healthcare systems, among others, as well as different modalities articulated for the care of the older adults. In this process, the articulation of formal and informal public, private and community actors stands out.

## Resumo

Este artigo descreve e analisa diferentes atores de cuidados em itinerários terapêuticos de idosos diagnosticados com e sobreviventes da COVID-19 em vários territórios do Chile. A estratégia metodológica foi qualitativa com uma abordagem etnográfica, com foco nos itinerários terapêuticos de idosos com 75 anos ou mais, sobreviventes da COVID-19, no período entre 2020 e 2021. Foi realizado o trabalho de campo em duas regiões, analisando experiências diferenciais nos processos de cuidado em áreas urbanas e rurais. Foram aplicadas análise de conteúdo narrativo e construção de histórias etnográficas sobre os itinerários terapêuticos de idosos e de seus cuidadores. Os resultados evidenciam a diversidade e complexidade dos atores envolvidos na trajetória do cuidado no contexto da infecção por COVID-19: família, vizinhos/as, amigos, sistema de saúde público e privado, entre outros, bem como diferentes modalidades em que são articulados para o cuidado dos idosos. Nesse processo destaca-se a articulação de atores públicos, privados e comunitários, formais e informais.

## Introducción

Este artículo presenta los resultados de una investigación que analiza la participación de distintos actores del cuidado en itinerarios terapéuticos de personas mayores sobrevivientes al COVID-19, en zonas urbanas y rurales en Chile. Considera como contextos, por una parte, el acentuado envejecimiento demográfico y la longevidad que presenta Chile y, por otra, las decisiones de atención en salud que toman las personas en contextos de crisis sociosanitaria.

Chile enfrenta un rápido envejecimiento poblacional, siendo uno de los países más envejecidos de América Latina y el Caribe. Entre 1950 y 2000, la proporción de personas mayores de 60 años creció del 6% al 16,8% ^1^, mientras la esperanza de vida aumentó de 52 a más de 80 años en 2020 ^2^. Se prevé que para 2050, las personas mayores de 64 años representarán el 25 % de la población ^2^.

El grupo de personas mayores de 60 años es heterogéneo y mayormente vulnerable en relación con el resto de la población, debido a la insuficiente respuesta del sistema de seguridad social, así como de las condiciones de vida y un mayor aislamiento social, entre otras razones ^3^. Las personas mayores son particularmente afectadas en situaciones de emergencias, desastres y crisis sociosanitarias; sus necesidades en estas situaciones son frecuentemente no abordadas, o abordadas en forma parcial y poco oportuna ^4^.

Durante la crisis sociosanitaria provocada por la pandemia por COVID-19, a nivel mundial se catalogó a la población de personas mayores como grupo de riesgo, al verse afectada de manera desproporcionada en mortalidad ^5^. Los sistemas de atención primaria de salud estaban sobreexigidos y su capacidad de respuesta mermó por la crisis, aumentando el riesgo de enfermar gravemente y morir ^6^. Esto implicó una presión y reorganización de los cuidados al interior de los grupos familiares.

Chile no fue la excepción a esta tendencia, concentrándose el 82,5% de las 57.656 muertes (sospechosas y confirmadas) causadas por COVID-19, en el grupo de mayores de 60 años (registradas al 4 de mayo de 2022). Estas cifras demuestran desafíos preexistentes enfrentados por las personas mayores en el acceso a servicios de salud, atención y cuidados ^7^, así como debilidades de los sistemas de salud para atender sus necesidades en contextos de crisis sociosanitaria, revelando brechas existentes en políticas, sistemas y servicios de salud ^6^. Ello debe entenderse, además, en el contexto de rápido envejecimiento poblacional y creciente prevalencia de enfermedades crónicas al que el sistema de salud chileno se ha visto enfrentado especialmente en la última década ^8^.

En Chile, la atención primaria de salud (APS) es la puerta de entrada al sistema, incorporando un modelo de atención integral territorializado y es el punto de enlace entre comunidad y sistema de salud ^9^. La pandemia por COVID-19 tensionó la resiliencia de los sistemas de salud en su capacidad de respuesta, adaptación, integración y participación de las personas y comunidades.

Las medidas sociosanitarias estatales durante el 2020 para la población mayor estuvieron centradas en el confinamiento obligatorio para mayores de 75 años, intervenciones psicosociales remotas, promoción de salud y elaboración de protocolos orientados al manejo y prevención de contagio. Estas acciones buscaban disminuir los efectos psicosociales y riesgos de contagio ^10^.

Desde la población, se tomaron distintas decisiones frente a la enfermedad, lo cual puede ser analizado con el concepto de itinerario terapéutico. Su análisis se centra en “*...todas las actividades desarrolladas por los individuos en la búsqueda de tratamiento por una dolencia o aflicción*” ^11^ (p. 30), a lo largo de un recorrido específico, durante el cual intervienen diferentes actores y redes sociales ^12^. En este itinerario, los actores pueden articularse o intervenir de manera independiente, a lo largo de la trayectoria que viven las personas durante el proceso de contagio, enfermedad y tratamiento. Se ha destacado que el análisis de los itinerarios terapéuticos es una excelente estrategia para conocer/comprender las prácticas de diversos grupos orientadas a resolver los problemas de salud, particularmente de sujetos más vulnerables ^13^.

Se han detectado diversos actores que participan en los itinerarios terapéuticos, individuales y colectivos; formales e informales ^12^ ^,^ ^13^. Desde la perspectiva de los cuidados, algunos/as autores/as retoman la propuesta de Razavi ^14^ del diamante de cuidados para analizar las políticas, así como los actores ^15^ ^,^ ^16^, la cual seguimos en este artículo para referirnos a aquellos que pueden intervenir a lo largo del itinerario terapéutico.

La identificación y análisis de los actores locales, relaciones y procesos que convergen con el sector salud es clave, pues son fundamentales para proveer servicios de calidad, cuidados centrados en la persona, construir sistemas de salud resilientes y fortalecer la resiliencia comunitaria ^17^. El involucramiento activo de la comunidad muestra que la acción y respuesta a situaciones de crisis, no solo debe descansar en la resiliencia de los sistemas de salud, sino también confiar en la participación comunitaria y en los cuidados que proveen diferentes actores para prevenir y manejar este tipo de situaciones ^18^.

## Métodos

Se usó un diseño cualitativo descriptivo ^19^ con enfoque etnográfico ^20^ que permitió reconstruir los itinerarios terapéuticos de personas mayores de 75 años contagiadas por COVID-19. La investigación se desarrolló en dos zonas de Chile. La Región Metropolitana por la alta concentración de servicios de salud y acceso a recursos de manera centralizada, al ser la capital del país. Y la Región de Los Lagos por presentar un desmembramiento territorial - con presencia de ríos, lagos, fiordos e islas - que incide en las dinámicas de su población ^21^, así como en el acceso heterogéneo y desigual a los servicios públicos de salud.

La muestra fue de tipo teórica-intencional, siguiendo la estrategia de casos ^22^, realizándose un constructo teórico de estudio, seleccionando unidades de muestreo de acuerdo con perfiles definidos conceptualmente. La muestra fue de seis personas mayores de 75 años que sobrevivieron al COVID-19, contagiadas durante los años 2020 y 2021, y que habitaban en contextos urbanos y rurales. Ello posibilitó obtener diversidad para cada territorio respecto a los procesos de atención de población mayor durante la crisis. El trabajo de campo se desarrolló entre septiembre de 2023 y enero de 2024.

**Figura 1:**
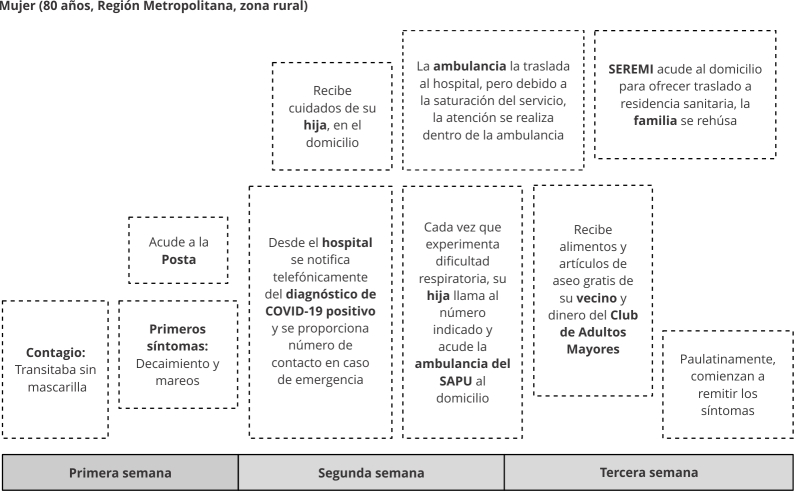
Itinerario terapéutico de actores.

SAPU: Servicio de Atención Primaria de Urgencia; SEREMI: Secretaría Regional Ministerial de Salud. Fuente: elaboración própria.

**Figura 2:**
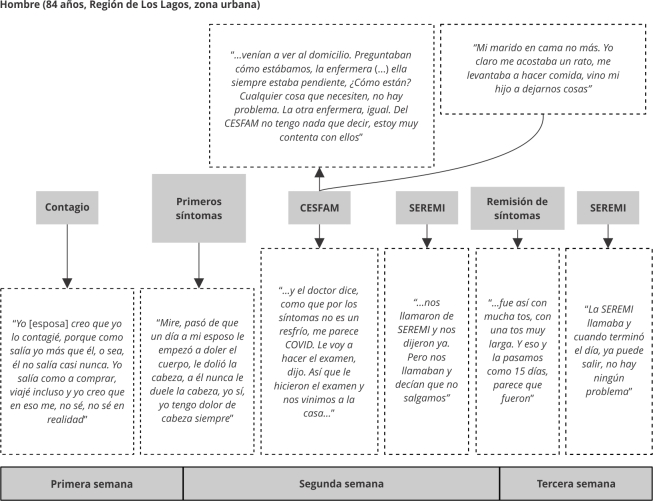
Itinerario terapéutico narrativo.

CESFAM: Centros de Salud Familiar; SEREMI: Secretaría Regional Ministerial de Salud. Fuente: elaboración própria.

Las técnicas de producción fueron entrevistas cualitativas ^23^ y observación etnográfica. El material producido fue grabado en audio y transcrito; de manera complementaria se tomaron notas de campo. Se realizó un análisis de contenido narrativo ^22^ con el apoyo del software Atlas.ti versión 23 (http://atlasti.com/), en un proceso inductivo de codificación abierta. Los itinerarios terapéuticos de los casos de estudio fueron reconstruidos con la plataforma Miro (https://miro.com). Este tratamiento de la información permitió reflejar de manera gráfica y global la diversidad de itinerarios terapéuticos, elaborándose dos tipos: (a) itinerario terapéutico de actores, con énfasis en la temporalidad del proceso de enfermedad, identificando hitos, actores involucrados e intervenciones (Figura 1) y, (b) itinerario terapéutico narrativo, con énfasis en la dimensión subjetiva, emocionalidad, acciones y toma de decisiones del proceso, incorporando las voces de personas mayores (Figura 2).

Las Figuras 1 y 2 fueron seleccionadas como ejemplo de los itinerarios terapéuticos y su versión gráfica a través del programa Miro. Buscamos dos casos diversos en términos de género y territorio. En la Figura 1 se destaca la diversidad de actores involucrados en el itinerarios terapéuticos y en la Figura 2, además de los distintos actores, destacamos narraciones significativas de la persona entrevistada en relación a los actores.

El acceso e identificación de los casos fue a través de los equipos profesionales del sistema primario de salud. La investigación contó con la aprobación del Comité de Investigación en Seres Humanos de la Facultad de Medicina de la Universidad de Chile (protocolo nº 215-2022), y del Comité de Ética de los Servicios de Salud Metropolitano Central (protocolo nº 200/2023), donde se desarrolló el trabajo de campo. Todas las personas entrevistadas en este estudio autorizaron su participación a través de la firma de consentimiento informado.

## Resultados

### Actores del cuidado a nivel familiar

A partir del análisis realizado, observamos que la familia es uno de los actores más relevantes en los cuidados a lo largo del itinerario terapéutico, tanto en el contexto urbano como rural, así como en el ámbito doméstico y en la gestión y acompañamiento del proceso de hospitalización, en caso de existir. La familia no solamente refiere a las personas que cohabitan con el enfermo/a, sino que se advierte la relevancia de las relaciones y redes familiares, más allá de la presencialidad.

Al interior del núcleo doméstico, son las hijas y nietas quienes participan en mayor medida en el acompañamiento, gestión y cuidado de las personas mayores enfermas.

La gestión de estos cuidados se da en distintos espacios en los que transcurre el itinerario, que puede desarrollarse en su totalidad en el hogar, o transitar a centros de salud. La diversidad de espacios, así como la distancia entre hogares y lugares de atención, incorporan complejidad y dificultades a las acciones de las familias para la atención de las personas contagiadas. Ello es más crítico en las zonas rurales de nuestro estudio, donde la atención médica se ubica en centros públicos más lejanos al domicilio.

“*Y cuando mi papá presentó síntomas, yo le sugerí que vayamos a algún SAPU* [Servicio de Atención Primaria de Urgencia]*, porque la posta de acá creo que estaba cerrada...*” (nieta, sector rural).

Asimismo, destacamos la variedad de tiempos y momentos en que transcurren las acciones desplegadas por familiares durante el itinerario terapéutico. Pueden constituirse en acciones acotadas como llamadas telefónicas a los servicios de salud, pero generalmente suponen una alta dedicación temporal, que pueden ser horas, días, semanas o incluso meses. Si bien estas acciones no siempre son permanentes, implican una atención y preocupación constante por parte de los familiares.

Es relevante resaltar que una parte importante de las gestiones con las instituciones es llevada a cabo por la familia, específicamente por las hijas y en menor medida, las nietas, quienes participan de distintas acciones durante los itinerarios terapéuticos. Ellas establecen el diálogo más formal y oficial con los equipos de salud, así como las gestiones cotidianas de atención y cuidado. Las hijas/os participan o toman decisiones en relación con las atenciones de salud durante el proceso. Al ser personas mayores, sus hijos/as son generalmente adultos/as, por lo que cuentan con autoridad para participar de las disposiciones familiares en relación con sus padres y madres.

“*...me avisaron que no tenía COVID, la hija iba todos los días...*”. Entrevistador: “*¿Y acá quién era su referente allá en la familia? ¿Su hija era la que siempre estaba preguntando?*”. “*Mi hija, la de en medio, era la vocera, era la tutora*” (hombre, 78 años, sector urbano).

Como advertimos en algunos de nuestros casos, las relaciones familiares se extienden más allá de la filiación, participando otros miembros, incluyendo a parientes políticos.

“*Y llegó una niña, una enfermera que trabajaba ahí, que era compañera de una sobrina. Y ella fue a preguntar por mí. Mi sobrina le dijo que tratara de conseguir, si me podían ubicar y todo, porque mi familia no sabía. Y ella me sacó una foto para que me vieran que yo estaba en el hospital y estaba ya un poquito lúcida*” (mujer, 78 años, sector urbano).

“*Realmente no tenía tanta fiebre. Así que, como a las 7 de la mañana, mi yerno dijo, ‘no, ya’, dijo, ‘llevémoslo para el hospital, a alguna parte, no, cómo va a estar así. Si está mal, está enfermo’...*” (hombre, 79 años, sector urbano).

El rol de articulación de los miembros de las familias, con los distintos actores del cuidado formal e informal, son relevantes a la hora de enfrentar diversas acciones y decisiones que se toman en el trayecto del itinerario terapéutico. Dicha articulación es facilitada, en ciertos casos, por el acceso a tecnología y medios de comunicación, que permiten establecer contacto con servicios médicos y asistir a los familiares enfermos, aun cuando no se conviva en la misma casa o existan distancias geográficas.

“*Mi hija llamaba la ambulancia y me llevaban. Porque estaban asustadas*” (mujer, 80 años, sector rural).

Asimismo, hay una preocupación constante y seguimiento del estado de salud de las personas contagiadas. Al respecto, como ya hemos detallado, son principalmente las mujeres de la familia quienes asumen dichas labores cotidianas.

“*Sí, estaban asustadas y todos los que estábamos en la casa. Incluso mi hija se acostaba conmigo porque le daba miedo dejarme sola*” (mujer, 80 años, sector rural).

“*Me trajeron una sopa, me la trae mi hija y yo le digo, ‘no, no quiero, llévate el plato’ y le dice mi nieta: ‘A ver, pásame el plato para acá y ándate para afuera’. Y viene y me obliga a comer unas dos cucharadas. Me obligó a comer*” (mujer, 80 años, sector rural).

### Actores del cuidado a nivel comunitario

En el ámbito comunitario se identificaron diversos actores que desempeñaron un rol relevante en la provisión de cuidados durante la crisis sociosanitaria. Tales actores, tanto individuales como colectivos, se ubican en tres niveles diferentes: las redes locales más cercanas a los hogares, organizaciones comunitarias territoriales e institucionalidad local.

Dentro del espacio comunitario más próximo a los hogares, se destaca la actuación de vecinas/os, que contribuyen llevando medicamentos a quienes se encuentran en aislamiento luego de ser diagnosticados/as con COVID-19 y también en el traslado de personas mayores hacia centros de vacunación.

“*Tenía que comprar los remedios. Así que venía un vecino que tengo que es muy amoroso... él iba a comprar los remedios porque él tiene auto*” (mujer, 80 años, sector rural).

Asimismo, los/as propietarios/as de pequeños comercios locales articulan sistemas de despacho a domicilio de alimentos y artículos de aseo, para evitar que las personas mayores, definidas como grupo de riesgo, se expongan al contagio al salir a comprar.

“*Ellos me prestaron mucha ayuda, los dueños del almacén me venían a dejar acá las cosas, que no me preocupara, que después arreglábamos.* (...) *porque nadie podía salir de acá. Entonces venían personas, me traían cosas de regalo; me traían verduras. El primer día me trajo una manga de confort* [papel higiénico] *y cloro*” (mujer, 80 años, sector rural).

En general, los vínculos entre las personas mayores y estos actores tienen un carácter previo, más espontáneo y flexible, en comparación a aquellos que establecen con las organizaciones comunitarias e instituciones del gobierno local. Cabe destacar que algunos de estos pequeños comercios desarrollaron sus propios mecanismos de trazabilidad mediante el registro de quienes acudían a comprar, de manera que, si se producía un contagio, era factible rastrear a otros/as posibles afectados/as.

“*En los negocios también tenían un sistema bien bueno allá* (...)*. Ahí tenían un cuaderno que uno iba a comprar y quedaba registrado* (...) *Entonces ella decía, por si alguien se enferma en ese día que digan, ‘hay una persona enferma con COVID’, ya voy a saber que todos estuvieron aquí*” (hombre, 84 años, sector urbano).

Por otra parte, las organizaciones comunitarias territoriales como Juntas de Vecinos y Clubes del Adulto Mayor realizan aportes directos a los hogares, entregando dinero y productos de primera necesidad, como alimentos, artículos de higiene y gas, para calefaccionar y cocinar.

“*Sí, eso de la Muni* [municipio] *y después tuvimos ayuda por la Junta de Vecinos.* (...) *También nos llegó una caja de mercadería de la Junta de Vecinos*” (mujer, 78 años, sector urbano).

“*Sí, del Club que yo estaba, Club del Adulto Mayor también, me dieron plata*” (mujer, 80 años, sector rural).

De manera similar, la institucionalidad local -principalmente municipios-, también se enfoca en la dimensión material de los cuidados ^24^ proporcionando víveres y productos de aseo.

En el marco de los itinerarios terapéuticos, particularmente en aquellos casos que requirieron hospitalización, emerge una forma de comunidad inesperada, lo que en esta investigación se ha denominado comunidad intrahospitalaria. Este concepto alude a una red de cuidados que se va tejiendo dentro del contexto hospitalario entre pacientes o bien, entre pacientes y personal de salud que ya ha pasado por la experiencia de enfermedad por COVID-19. Esta red ejerce un rol de contención emocional, aspecto particularmente relevante en un contexto de incertidumbre, el aislamiento de los vínculos afectivos más cercanos y el malestar producto de la enfermedad. También, permite informar al personal de salud cuándo un paciente necesita asistencia, en los casos en que este debido a su condición no puede hacerlo por sí mismo. Y, por último, facilita la comunicación por vía telefónica con las familias, cuando no existía posibilidad de recibir visitas y los mecanismos formales de comunicación con el exterior resultaban insuficientes.

“*Una señora que conocí yo aquí en el hospital, y fuimos las dos trasladadas para allá, y quedamos en la misma sala de la UCI* (...) *la señora se quedó con el celular. Y me dice, ‘¿Juana tu familia sabe dónde estás?’, ‘No’, le dije yo, ‘sabes que no tienen ni idea del día que me hospitalizaron’, y me dice, ‘¿quieres avisarle?’ Claro, ella estaba al frente, yo no podía hablar con mi familia, y le digo, ‘ya márcame este número, por favor’. Le doy el número a la Paula, y contestó mi hija en la noche, y mi hija le dice a ella, ‘¿y usted por qué tiene el número de mi mamá?’ La Paula le dice,’ es que somos hermanas de COVID’ y le dijo, ‘estamos las dos en la misma pieza’. Y yo de acá le grito, le digo, ‘soy yo hija, si es verdad’, le digo, ‘estamos las dos juntas’, y ella me dice, ‘¡estás viva mamá!’, dice, se siente el grito. Ahí supieron que yo estaba en el Hospital*” (mujer, 78 años, sector urbano).

### Actores del cuidado a nivel institucional

Los actores institucionales están representados por los Centros de Salud Familiar (CESFAM), SAPU y los hospitales con sus servicios de hospitalización y urgencia. Además de aquellas instituciones ligadas a las medidas sanitarias implementadas, como son las llamadas residencias sanitarias o programas de atención domiciliaria, seguimiento telefónico, y traslados. A nivel nacional la Secretaría Regional Ministerial (SEREMI) de Salud fue la institución responsable de la trazabilidad y de fiscalizar el seguimiento de las medidas de confinamiento y cuarentena.

A nivel global, las personas mayores se vieron bastante afectadas por la mortalidad por COVID-19, por lo mismo, fueron un grupo poblacional objetivo de políticas y medidas sanitarias específicas. Estos actores institucionales adquieren mayor protagonismo y presencia en determinados momentos de los itinerarios terapéuticos de las personas mayores entrevistadas.

La confirmación del diagnóstico se realiza a través de una prueba (PCR; proteína C reactiva) que llevan a cabo los servicios de salud, y suele marcar el inicio del itinerario terapéutico en pandemia.

“*Vino una enfermera para acá y me hicieron el examen a mí. Ya al rato ya llegó ella..., me llama y me dice, ‘no se mueva de la casa porque están los dos contagiados* [ella y su esposo]” (esposa, sector urbano).

Para el caso de las zonas rurales, algunas pruebas se realizaban desde la posta rural en los domicilios de las personas mayores, por las dificultades de movilidad y traslado en territorios más aislados, como ha sido evidenciado en otros países ^25^. En estas situaciones, los servicios prestados por el personal de salud de postas rurales, asumen un rol importante y de relación directa con las familias que tenían a personas mayores con síntomas. Al haber brotes en las unidades domésticas, son los profesionales de la salud quienes se trasladan a los hogares para hacer las pruebas, dar indicaciones terapéuticas y de aislamiento.

“*En ese momento tuvimos como cierta sospecha de que podía ser COVID. Y después, en un par de días, mi papá igual empezó a presentar síntomas. Llamamos al paramédico de la posta de acá, diciéndole todos los síntomas que teníamos. Y que mi abuela era de preocupación porque era paciente con asma. Él nos sugirió que vayamos a un centro asistencial, a un SAPU. Lo llamó por radio y al tiro vinieron los médicos*” (nieta, sector rural).

La agencia de las propias personas mayores también está presente en la experiencia y relación con los actores institucionales y familiares. Tal es el caso de una entrevistada que habitaba en sector rural, y que nunca aceptó ser hospitalizada; era trasladada al hospital para recibir atención, sin embargo, siempre volvía a su hogar.

“*Mi hija llamaba a la ambulancia, porque estaban asustadas. Venía la ambulancia, yo iba más o menos ahogada y me ponían oxígeno, me hacían exámenes, me sacaban sangre, porque andaba equipada con todo... me llevaban al hospital, a veces me tenían como más de una hora en la ambulancia afuera porque estaba muy saturado*” (mujer, 80 años, sector rural).

Las hospitalizaciones y las derivaciones de las personas mayores a residencias sanitarias son responsabilidad y decisión de la institucionalidad de salud. La hospitalización en centros especializados solo se realiza al agudizarse los síntomas. Vale decir que, la atención terciaria de salud se presenta cuando los cuidados deben realizarse en una institución del sistema médico y los tratamientos necesarios tienen mayor complejidad, como, por ejemplo, ser intubados o necesitar atención especializada y constante. Esta experiencia de hospitalización y atención especializada, las personas mayores la viven y significan desde la emocionalidad, tal y como se describiera en el apartado anterior, generando un sentido de mayor intimidad y cercanía al estar institucionalizados.

“*Claro yo siempre a modo de gratitud, como fueron en el hospital, la atención que me brindaron, la dedicación fue hermosísima... al final me salvaron la vida. La doctora iba a verme todos los días, y cuando me* [dieron el alta] *me abrazó*” (hombre, 78 años, sector urbano).

Los actores institucionales mantienen una interacción constante con las familias, quienes se contactan con los centros especializados para la atención de sus miembros mayores, tanto en zonas urbanas como rurales. En las fases finales del itinerario terapéutico, como el seguimiento y monitoreo posterior de personas sobrevivientes, se realizan llamadas telefónicas diarias o visitas del personal de salud a los domicilios hasta que se autoriza el control de las personas mayores nuevamente en los CESFAM.

“[Preguntaban] *...nos decían que no salgamos a ningún lado. Era para lo que más llamaba la señora y decía, ‘no tienen que salir a ningún lado, permanezcan en su casa’. La SEREMI llamaba y cuando terminó el día* [de cuarentena]*, ya puede salir, no hay ningún problema*” (hombre, 84 años, sector urbano).

## Discusión y palabras finales

Este artículo se propuso analizar la participación de distintos actores del cuidado en los itinerarios terapéuticos de personas mayores sobrevivientes al COVID-19, en zonas urbanas y rurales en Chile. Los hallazgos muestran que existen distintos actores relevantes, destacando tres de ellos en todos los casos analizados: familiares, comunitarios e institucionales. Respecto del ámbito familiar, como ha sido destacado en otros estudios sobre itinerarios terapéuticos ^26^ ^,^ ^27^, son particularmente las mujeres de las familias quienes establecen vínculos entre las personas mayores enfermas y el sistema de salud, movilizando recursos, trasladando a sus familiares, llamando a los servicios médicos públicos y/o privados, así como dialogando -en caso de ser posible- con el personal de salud. La familia participa de lo que Oliveira et al. ^28^ denominan sistema informal en el sistema de cuidados de personas enfermas, a lo largo del itinerario terapéutico y es central en la red de cuidados. Efectivamente, nuestros hallazgos profundizan lo que otras investigaciones han mostrado respecto de la responsabilidad principalmente femenina de los cuidados, tanto en la gestión y organización de estos, como en prácticas específicas y concretas durante distintos momentos que transcurren desde que las personas inician con síntomas ^29^ ^,^ ^30^. A pesar de ello, y en concordancia con lo que indican Passerino & Zenklusen ^29^, esta responsabilidad y participación femenina en los cuidados, no es reconocida en el enfrentamiento de la pandemia de COVID-19.

Por su parte, encontramos que los actores comunitarios, formales e informales, colaboran de manera complementaria a la red familiar y de salud -en coherencia con lo indagado en Brasil por Giovanella et al. ^31^- particularmente en contexto de emergencia y de acuerdo con vínculos previamente establecidos.

Un hallazgo relevante de este estudio es la presencia de lo que hemos denominado una “comunidad intrahospitalaria”, que apoya los cuidados en un escenario de escasa información durante la emergencia.

En este contexto, los resultados encontrados muestran una diversidad de recursos materiales, simbólicos, emocionales, que están presentes y circulan entre los distintos actores del itinerarios terapéuticos, como ha destacado Brage ^12^ en su análisis de itinerarios terapéuticos en Argentina.

Nuestro estudio destaca, además, la identificación de dinámicas y contextos territoriales, que favorecieron la generación de prácticas de cuidados desde lo público y colaborativo ^32^. La presencia de actores comunitarios vinculados con los cuidados es transversal durante la pandemia, como ha sido destacado por Torres-Hernández et al. ^33^ también en España, y no se restringe a un momento específico de los itinerarios terapéuticos. La población mayor fue uno de los grupos con más restricciones sociosanitarias, debiendo permanecer confinados y aislados por largos períodos ^34^, por lo que estos apoyos y cuidados que contribuyen al sostenimiento de la vida cotidiana resulten tan significativos.

No se establecen diferencias importantes en cuanto a los cuidados comunitarios en sectores urbanos y rurales. Se observa que en los casos en que el acompañamiento y tratamiento se lleva a cabo principalmente en el domicilio, se intensifica la carga de cuidados familiares, mas no hay cambios sustanciales en su dimensión comunitaria, dando cuenta de la predominancia de una lógica familista e individualista de los cuidados, que siguen en contextos de crisis sociosanitaria, constituyendo un asunto que se resuelve fundamentalmente en el ámbito privado ^35^.

Por su parte, los actores institucionales de salud participan a lo largo del proceso, demandados por las familias, quienes participan en distintos momentos del proceso de enfermedad y tratamiento e intervienen en el marco de los recursos disponibles y la organización en las distintas etapas de la pandemia.

Este estudio analizó una muestra de casos provenientes de dos regiones del país, lo que podría limitar la diversidad de actores considerados en los itinerarios terapéuticos de personas mayores. Además, se incluyeron únicamente casos de personas mayores residentes en la comunidad, lo que sugiere que, para quienes viven en residencias u otras condiciones habitacionales, los actores de cuidado involucrados en sus itinerarios terapéuticos podrían diferir de los descriptos. A pesar de estas limitaciones, el estudio aporta elementos clave sobre los actores locales, las relaciones y los procesos durante la pandemia de COVID-19. Entre sus hallazgos se destaca la emergencia de una comunidad intrahospitalaria de redes de cuidado conformada por personas afectadas por COVID-19, así como la centralidad de las familias, y especialmente de las mujeres, como pilares fundamentales del cuidado, incluso en contextos de crisis.
